# Gastric Glomus Tumor Mimicking a Neuroendocrine Tumor: A Case Report Highlighting Diagnostic Pitfalls

**DOI:** 10.70352/scrj.cr.26-0574

**Published:** 2026-10-14

**Authors:** Susumu Ohwada, Shinji Sakurai, Yuhei Nakano, Takayuki Aiba, Toshiya Takemura, Hideki Matsuyama

**Affiliations:** 1 Center of Gastroenterology and Oncology, IMS Ota Chuo General Hospital, Ota, Gunma, Japan; 2 Department of Pathology, Gunma Chuo General Hospital, Maebashi, Gunma, Japan

**Keywords:** gastric glomus tumor, neuroendocrine tumor, gastrointestinal stromal tumor, subepithelial lesion, laparoscopic and endoscopic cooperative surgery, immunohistochemistry

## Abstract

**INTRODUCTION:**

Gastric glomus tumors (GGTs) are rare mesenchymal neoplasms arising from the modified smooth muscle cells of the glomus body. Their subepithelial location and pronounced hypervascularity overlap considerably with gastrointestinal stromal tumors (GISTs) and neuroendocrine tumors (NETs) on imaging and in small biopsy specimens, making an accurate preoperative diagnosis challenging; as a result, such lesions are frequently diagnosed after surgical resection.

**CASE PRESENTATION:**

A 38-year-old asymptomatic woman was found to have a 25-mm well-circumscribed, hypervascular submucosal lesion with a small central depression and minute ulceration in the upper gastric body during a routine health-screening esophagogastroduodenoscopy (EGD). Endoscopic ultrasonography (EUS) demonstrated a heterogeneous hypoechoic mass arising from the third sonographic layer without evidence of muscularis propria invasion. Contrast-enhanced CT showed a hypervascular lesion with no regional lymphadenopathy or distant metastasis. Conventional forceps biopsies targeting the minute ulceration, evaluated with hematoxylin and eosin (H&E) staining alone, were interpreted as consistent with a NET, leading to a provisional diagnosis of a type III gastric NET. Because of the diagnostic uncertainty surrounding the NET diagnosis, and because the EUS and CT showed no evidence of deep or nodal spread, the patient underwent laparoscopic and endoscopic cooperative surgery (LECS) with a local full-thickness gastric resection rather than upfront gastrectomy with lymphadenectomy, and the defect was repaired through a protective mini-laparotomy. Definitive histopathology demonstrated uniform round-to-spindle cells arranged in a perivascular sheet pattern within a myxoid stroma. Immunohistochemistry (IHC) showed focal positivity for α-smooth muscle actin (α-SMA) and negativity for KIT (CD117), CD34, chromogranin A, synaptophysin, desmin, and the S-100 protein, confirming a GGT. All surgical margins were negative, and no recurrence has been observed over 5 years of follow-up.

**CONCLUSIONS:**

A GGT must be included in the differential diagnosis of hypervascular gastric subepithelial lesions (SELs), including those with a nested, NET-like architecture. H&E staining alone is insufficient for a definitive diagnosis; direct communication with the pathologist and comprehensive IHC profiling are indispensable. LECS is a favorable and effective organ-preserving strategy for localized gastric SELs when the diagnosis remains uncertain despite the preoperative evaluation.

## Abbreviations

α-SMA
: α-smooth muscle actin
EFTR
: endoscopic full-thickness resection
EGD
: esophagogastroduodenoscopy
EGJ
: esophagogastric junction
EUS
: endoscopic ultrasonography
GGT
: gastric glomus tumor
GIST
: gastrointestinal stromal tumor
H&E
: hematoxylin and eosin
HPF
: high-power field
IHC
: immunohistochemistry
LECS
: laparoscopic and endoscopic cooperative surgery
NET
: neuroendocrine tumor
SEL
: subepithelial lesion

## INTRODUCTION

GGTs are uncommon mesenchymal neoplasms originating from the modified smooth muscle cells of the glomus body, a specialized thermoregulatory arteriovenous anastomosis located predominantly in the dermis of the distal extremities.^1–3)^ Although the great majority of GGTs arise in the subungual region, visceral counterparts have been documented. GGTs constitute a particularly rare subset, accounting for fewer than 1% of all gastrointestinal mesenchymal tumors.^2,4)^

GGTs typically present as solitary SELs in the gastric antrum and are most often discovered incidentally during upper endoscopy.^1–5)^ Despite improvements in endoscopic and cross-sectional imaging, preoperative diagnosis remains challenging because GGTs share clinical, endoscopic, and radiological features with more common gastric SELs—namely, GISTs, NETs, leiomyomas, and neurofibromas.^2–6)^ Several reports have described a GGT mimicking a NET on EUS-guided or forceps biopsy^2,4–7)^; and in 1 series, GGT was similarly misclassified as GIST in 6 of 11 cases (55%).^7)^ Consequently, GGTs are frequently taken to surgical resection on the basis of imaging and biopsy findings alone, with the diagnosis confirmed only postoperatively, requiring histopathological and immunohistochemical evaluation of the resected specimen for definitive diagnosis.^3,5,7)^ Immunohistochemically, GGTs characteristically express α-SMA and are consistently negative for KIT (CD117), CD34, chromogranin A, synaptophysin, and S-100 protein.^2,3,8)^

Herein, we present a rare case of GGT initially misdiagnosed as a gastric NET on the basis of a preoperative biopsy evaluated without IHC. This case highlights 2 clinically relevant and insufficiently characterized issues. First, we examine the morphological features of the small biopsy specimen that led to the initial diagnosis of NET and whether retrospective assessment could have suggested GGT. Second, we address the therapeutic implications of this misdiagnosis. At this tumor size, a presumed type III gastric NET^9)^ would generally warrant consideration of gastrectomy with lymphadenectomy, whereas GGT can usually be treated by local excision. This case underscores the importance of recognizing this diagnostic pitfall and adopting an organ-preserving surgical strategy when the preoperative diagnosis remains uncertain.

## CASE PRESENTATION

A 38-year-old previously healthy woman underwent a routine upper gastrointestinal endoscopy as part of a health-surveillance program initiated because of a family history of esophageal carcinoma. She was entirely asymptomatic and denied dyspepsia, unintentional weight loss, hematemesis, or melena. Physical examination findings were unremarkable, and routine laboratory investigations—including a complete blood count and hepatic and renal function panels—were within normal limits.

EGD revealed a well-circumscribed, round, protuberant lesion with a small central depression and minute ulceration on the posterior lesser curvature of the upper gastric body (**Fig. 1A**). EUS using a 20-MHz radial probe (GF-UE290; Olympus Medical Systems, Tokyo, Japan) demonstrated a 25-mm heterogeneous hypoechoic mass arising predominantly from the third (submucosal) sonographic layer, with no evidence of invasion into the fourth (muscularis propria) layer (**Fig. 1B**). Contrast-enhanced CT demonstrated a well-defined hypervascular lesion, a pattern consistent with a hypervascular SEL, measuring approximately 25 mm in greatest dimension; no regional lymphadenopathy or distant metastasis was identified (**Fig. 2A** and **2B**). Endoscopic biopsy targeting the minute ulceration was performed using conventional forceps, as the lesion was directly accessible through its ulceration. EUS-guided fine-needle biopsy was not undertaken at this stage. Histological evaluation using H&E staining alone demonstrated solid-alveolar nests of cells with small, uniform nuclei and vacuolated, foamy cytoplasm, with focal trabecular arrangement and peripheral palisading—features interpreted as consistent with a well-differentiated NET. IHC was not performed, and a Ki-67 proliferative index and mitotic count were not assessed on the biopsy specimen. Serum gastrin was within the normal range (190 pg/mL; reference, <200 pg/mL), and no endoscopic evidence of atrophic gastritis was identified.

**Fig. 1:**
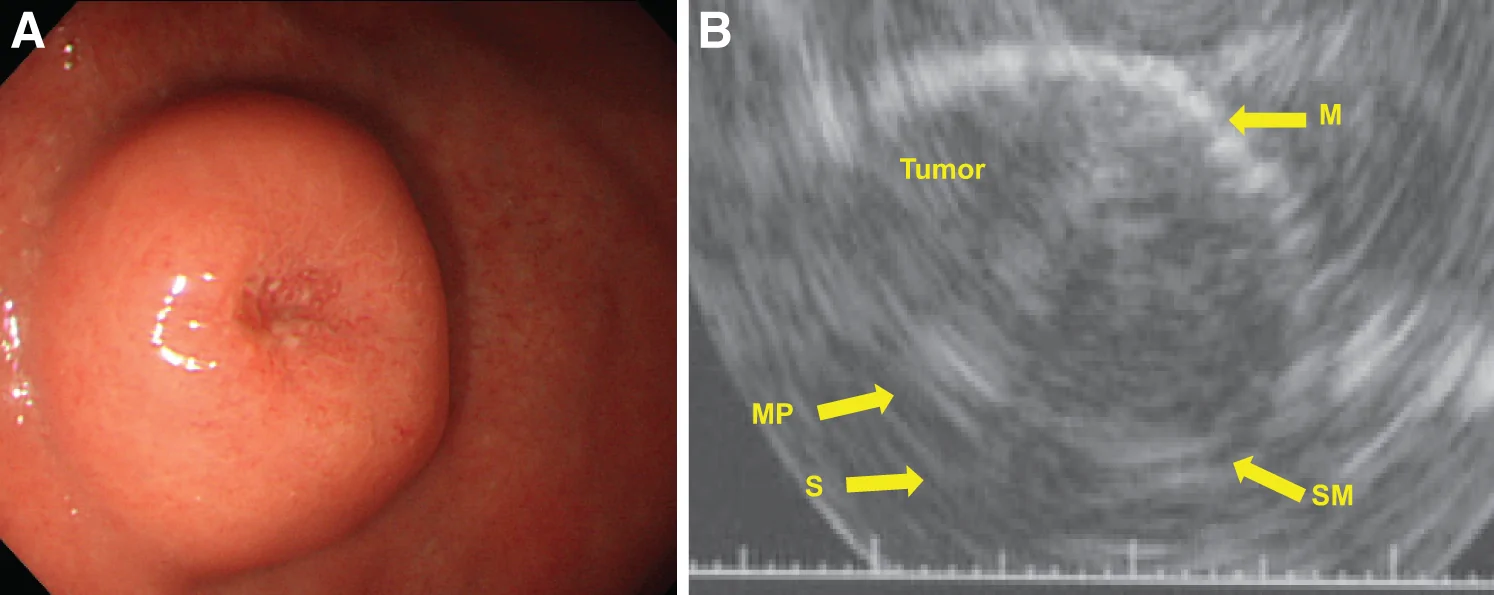
Endoscopic and endosonographic findings. (**A**) EGD reveals a well-circumscribed, round, protuberant lesion with a small central depression and minute ulceration on the posterior lesser curvature of the upper gastric body. The overlying mucosa is intact except at the depressed apex. (**B**) EUS (20-MHz radial probe) demonstrates a 25-mm heterogeneous hypoechoic mass arising predominantly from the third (submucosal) sonographic layer. EGD, esophagogastroduodenoscopy; EUS, endoscopic ultrasonography; M, mucosal layer; MP, muscularis propria layer; S: serosal layer; SM, submucosal layer

**Fig. 2:**
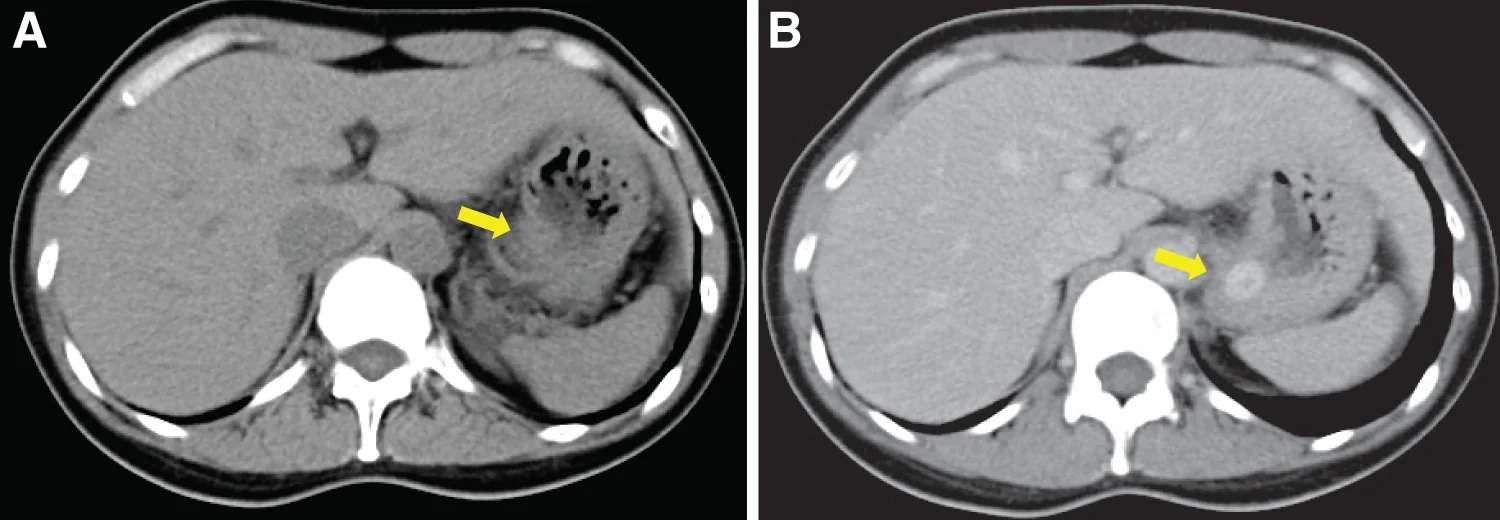
CT. (**A**) Axial plain CT image showing a mass in the posterior wall of the upper gastric body (arrow). (**B**) Axial contrast-enhanced CT image, obtained during the late arterial/portal phase, demonstrates a well-defined, homogeneously enhancing submucosal lesion measuring approximately 25 mm in greatest dimension (arrow).

Preoperative biopsy, EUS, and imaging findings suggested gastric NET type III,^9)^ which would warrant gastrectomy with lymph-node dissection according to the Japan Neuroendocrine Tumor Society (JNETS) guideline.^10)^ However, the endoscopic features—including reddish discoloration, central depression, and minute ulceration—were more suggestive of GIST, while the third-layer origin on EUS was not specific to NET. Given these diagnostic discrepancies, the risk of overtreatment with upfront gastrectomy, potentially requiring proximal or total gastrectomy with extensive lymph-node dissection, was considered substantial at the multidisciplinary conference. After informed consent and shared decision-making, LECS^11)^ was selected as the initial strategy, providing complete local excision with an intact specimen for definitive histopathological diagnosis while preserving the option of additional surgery based on the final pathology. We performed exposed EFTR^12)^ because the lesion was located in the upper gastric body near the EGJ. The resulting gastric wall defect was closed in 2 layers using continuous mucosal and interrupted seromuscular sutures through a protective mini-laparotomy, thereby minimizing luminal deformity and the risk of EGJ stenosis.^13)^ The total operative time was 125 minutes, and estimated blood loss was negligible. The postoperative course was uneventful, and the patient was discharged on POD 5 without complications.

Gross examination revealed a well-circumscribed, round, tan-purple to red subepithelial tumor measuring 25 mm in greatest dimension, with a hemorrhagic cut surface and focal overlying mucosal ulceration (**Fig. 3A**). The tumor was centered within the submucosa without macroscopic extension into the muscularis propria (**Fig. 3B**).

**Fig. 3:**
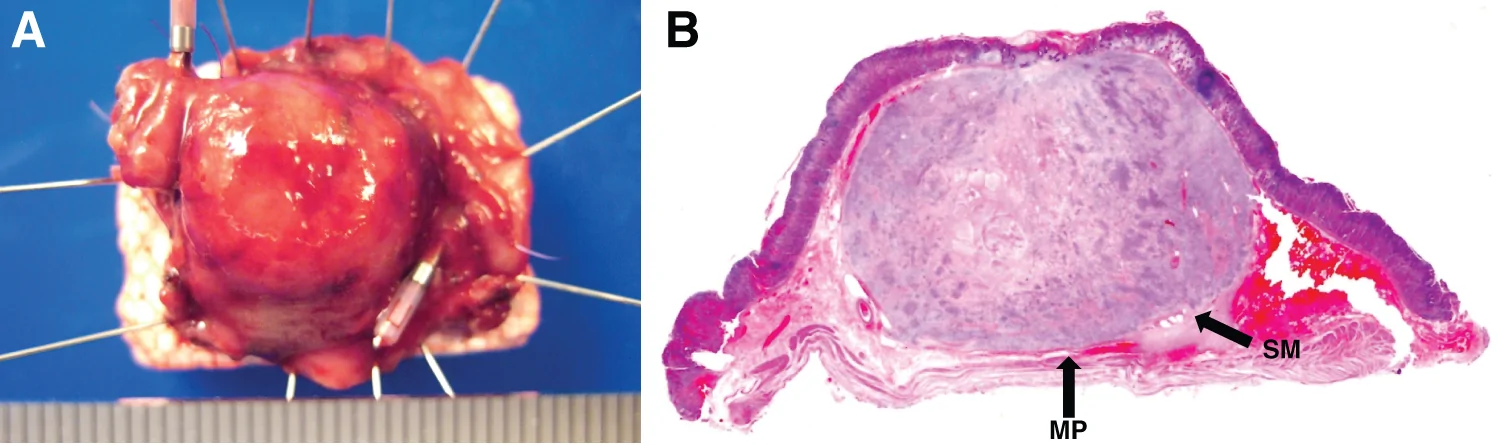
Gross and microscopic findings. (**A**) Gross specimen showing a well-circumscribed, round, tan-purple to red submucosal tumor measuring 25 mm in greatest dimension, with a hemorrhagic cut surface and focal overlying mucosal ulceration. Surgical margins are uninvolved. (**B**) Cut-surface loupe image demonstrating that the tumor is centered within the submucosa without extension into the muscularis propria.

Microscopically, the tumor comprised uniform spindle and ovoid cells arranged in prominent perivascular sheets, with focal peripheral palisading and interlacing bundles. Extensive myxoid stromal change was observed diffusely throughout the background matrix, separating the cellular cords (**Fig. 4A**). The initial pathological review favored GIST; however, an expert second-opinion review identified the characteristic perivascular arrangement of uniform glomus-like cells (**Fig. 4B**), prompting comprehensive IHC analysis. Tumor cells demonstrated focal cytoplasmic positivity for α-SMA (**Fig. 5A**) and were negative for KIT (CD117), CD34, chromogranin A, synaptophysin, desmin, and S-100 protein — apart from a few scattered KIT-positive mast cells (**Fig. 5B**). Mitotic activity was low (1 mitosis per 50 HPFs), and no necrosis or vascular invasion was identified. The Ki-67 proliferative index was approximately 2%–3%. These findings established the definitive diagnosis of GGT. All surgical margins were free of tumor (R0 resection). The patient remains alive without evidence of recurrence after 5 years of surveillance with periodic EGD and CT imaging.

**Fig. 4:**
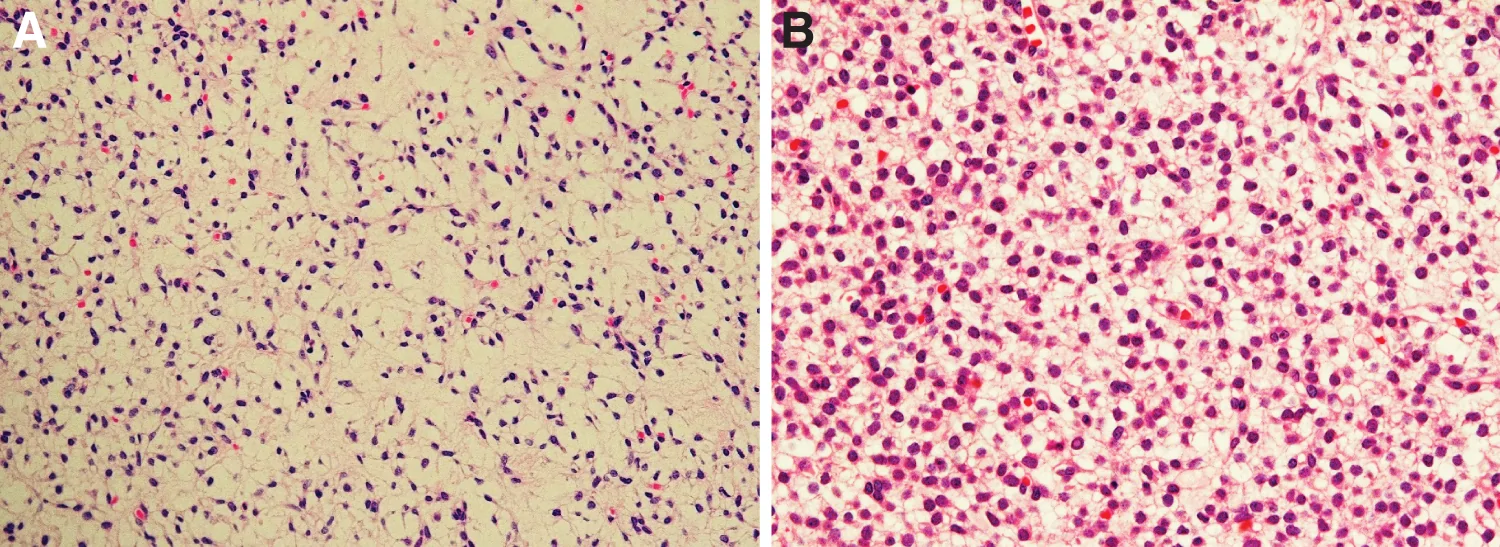
Histopathological features of the GGT. (**A**) Spindle-shaped to ovoid tumor cells exhibiting irregularly interlacing proliferation within a myxoid stroma. (H&E, ×200) (**B**) Sheets of round tumor cells arranged in a perivascular pattern, with sharply defined cell membranes, centrally located round to oval, uniform nuclei, delicate chromatin and inconspicuous nucleoli (H&E, ×200). GGT, gastric glomus tumor; H&E; hematoxylin and eosin

**Fig. 5:**
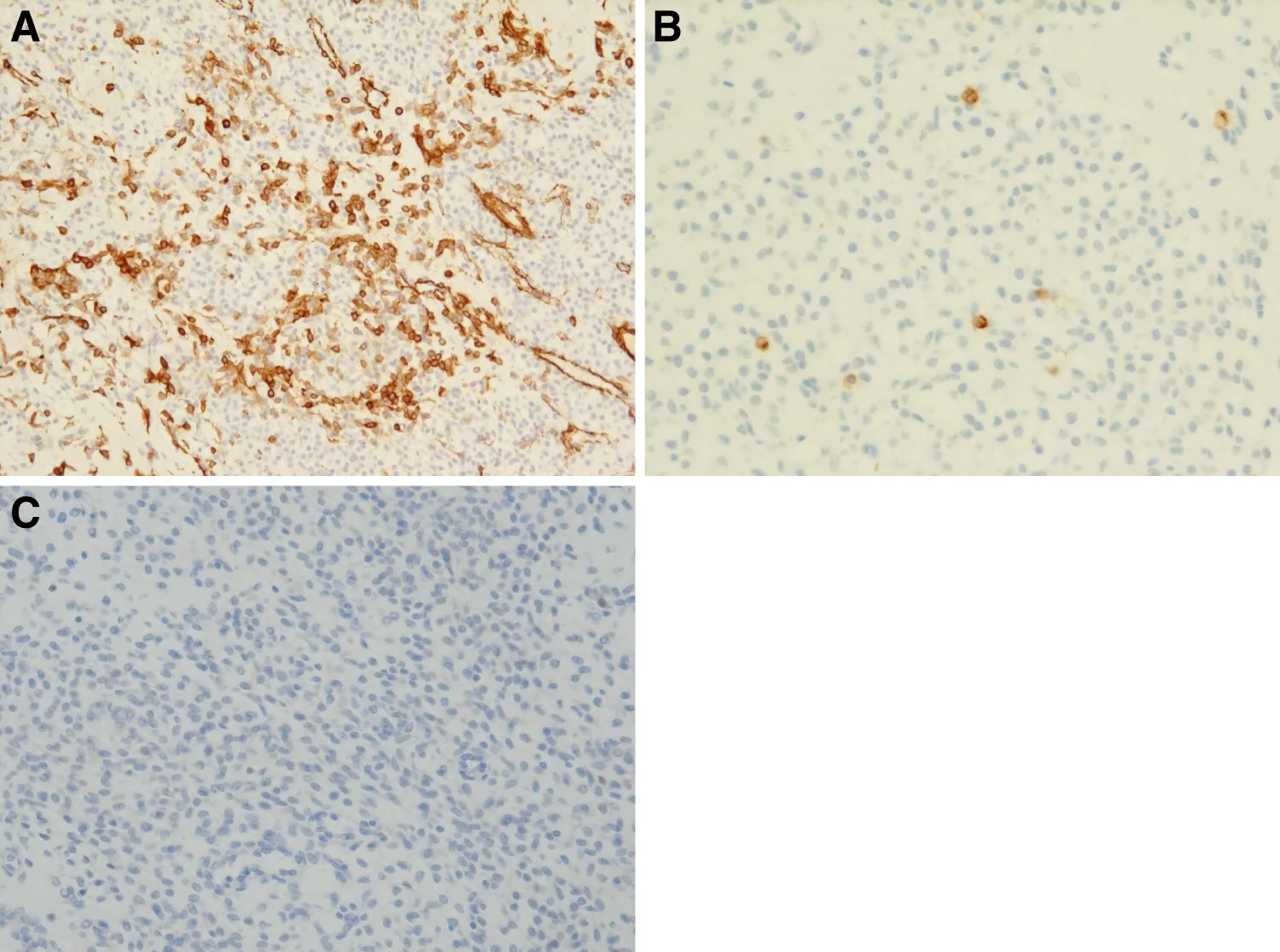
Immunohistochemical profile of the GGT (original magnification **A**, **C** ×100 **B** ×200; 4-μm formalin-fixed, paraffin-embedded sections). (**A**) Focal cytoplasmic positivity for α-SMA, confirming smooth muscle differentiation. (**B**) Absence of KIT (CD117) immunoreactivity, excluding GIST. Few KIT-positive mast cells are seen. (**C**) Negativity for S-100 protein, NET and peripheral nerve sheath tumor. α-SMA, α-smooth muscle actin; GGT, gastric glomus tumor; GIST, gastrointestinal stromal tumor; NET, excluding neuroendocrine tumor

## DISCUSSION

This case underscores an important lesson: when a multidisciplinary conference reveals discordant clinical and pathological findings, direct communication with the pathologist should be pursued. In retrospect, the absence of such communication at an earlier stage was a major regret in our management of this case. After the forceps biopsy specimen was reported as NET and the resected specimen was initially diagnosed as GIST, we ultimately requested an expert pathological review, which led to the definitive diagnosis of GGT.

This case illustrates 2 successive diagnostic errors: a preoperative misdiagnosis of NET based on H&E-only biopsy evaluation and an initial pathological impression of GIST on the resected specimen. These errors underscore the critical limitations of morphological assessment alone in the evaluation of gastric SELs. Difficulty distinguishing GGT from NET or GIST on preoperative sampling has been reported previously.^2–7)^ What this case adds is a detailed, feature-by-feature analysis of the reasons underlying the initial misinterpretation, an assessment of whether the diagnostic error could realistically have been avoided, and a demonstration of how the resulting diagnostic uncertainty was managed surgically. To our knowledge, these issues have not been addressed in comparable detail in previous reports. Retrospectively, the biopsy’s solid-alveolar and trabecular growth pattern, composed of uniform cells with round nuclei and vacuolated cytoplasm, closely mimics the nested or organoid architecture characteristic of NET G1/G2.^8)^ This morphological resemblance was the principal reason the diagnosis was missed. The peripheral palisading noted on the original biopsy interpretation, however, is also a recognized feature of GGT and is not specific to NET; had this been weighted differently at the time, the correct diagnosis might have been suspected preoperatively. In small biopsy samples, the defining perivascular architecture of glomus tumors is frequently not represented, rendering H&E-based classification unreliable.^2–4,7)^ The resected specimen additionally exhibited sheets and nests of monomorphic, small spindle-to-round cells embedded within extensive myxoid stroma, a pattern initially misinterpreted as GIST.^3,4,6,7)^

Both the biopsy and surgical specimens were initially evaluated at an external commercial pathology laboratory. One possible contributing factor to the diagnostic error is that additional IHC is not routinely performed in some commercial laboratories. Furthermore, a gastric GGT is extremely rare, and even pathologists at university hospitals or tertiary referral centers are unlikely to encounter it frequently; consequently, experienced pathologists may not readily consider GGT in the differential diagnosis of a gastric SEL, potentially contributing to diagnostic error. From a clinical perspective, when a gastric SEL is diagnosed without IHC analysis, clinicians should consider actively requesting such analysis to confirm the diagnosis; this approach may help reduce the risk of misdiagnosis.

The major differential diagnoses for gastric SELs include GIST, NET, leiomyoma, and, less commonly, neurofibroma. These entities can generally be differentiated by IHC staining, including GIST-associated markers such as KIT and DOG1, the smooth muscle marker desmin, and the neural/neuroendocrine markers S-100, synaptophysin, and chromogranin A. When these markers are negative, a rare entity such as glomus tumor should also be considered in the differential diagnosis. However, establishing a definitive diagnosis of a GGT based solely on biopsy specimens would likely be difficult even for an experienced pathologist.

The overlapping “small round cell” morphology and the potential for myxomatous stromal change are well-recognized sources of confusion among GGT, NET, and epithelioid-type GIST.^2–4,6,7)^ Appropriate IHC should therefore be considered in SEL biopsies whenever technically feasible because morphology alone may be insufficient for accurate classification. H&E staining alone may not always permit a confident diagnosis even in resected specimens, as illustrated by the initial GIST impression in this case; comprehensive IHC profiling is therefore important in both biopsy and surgical specimens. No residual biopsy material was available for retrospective IHC staining in this case, so it cannot be established with certainty that an IHC panel applied to the original small sample would have yielded interpretable, diagnostic staining; however, based on the panel obtained from the resected specimen, we consider it likely that α-SMA, desmin, KIT, S-100, chromogranin A, and synaptophysin staining performed on the biopsy—tissue permitting—would have raised the possibility of GGT preoperatively.

Endoscopically, GGT generally appears as a well-circumscribed, elevated mass with normal or ulcerated overlying mucosa.^2–4)^ Mucosal ulceration, documented in approximately 27% of cases, may result in clinically significant gastrointestinal bleeding.^3,5,7)^ The present tumor was atypical in its location — the upper gastric body near the EGJ, rather than the antrum. EUS has an advantage in identifying the layer of tumor origin,^14)^ no specific, definitive EUS, CT, or MRI findings exist for GGT. Marked hypervascularity and abundant intratumoral blood flow on these modalities may suggest the diagnosis but are insufficient for definitive preoperative diagnosis, as they are also frequently observed in GISTs and NETs.^3,4,6,7,14–16)^ This substantial imaging overlap among GGT, GIST, and NET underscores the importance of integrating endoscopic, radiological, and pathological findings; when the diagnosis remains uncertain, tissue acquisition with appropriate IHC analysis is essential for accurate classification and to avoid unnecessary extensive gastric surgery.

Surgical management of GGT is centered on complete local excision with negative margins.^7,17,18)^ Current guidelines—including those of the American Gastroenterological Association and the European Society of Gastrointestinal Endoscopy—do not provide a dedicated algorithm for GGT, and management strategies are therefore extrapolated from established best practices for NETs and GISTs.^17,18)^ Endoscopic submucosal enucleation is feasible and safe in selected cases.^19)^ However, LECS has emerged as a superior technique for gastric SELs, integrating precise endoscopic margin identification with laparoscopic full-thickness resection to minimize the blind margins associated with purely laparoscopic wedge resection.^11,13)^ LECS is particularly advantageous for preserving gastric volume and preventing luminal deformity, especially for lesions adjacent to the EGJ or pylorus.^11,13)^ Beyond the histological pitfall itself, this case provides an important practical lesson for the management of hypervascular gastric SELs with an inconclusive preoperative biopsy. Rather than committing to the more radical resection for a presumptive but IHC-unconfirmed type III gastric NET, an organ-preserving, margin-controlled local resection such as LECS can serve a dual purpose: definitive treatment of the lesion and acquisition of an intact specimen for accurate histopathological and IHC diagnosis. More extensive surgery can then be reserved for cases in which the final pathology demonstrates a clear oncological indication. This sequence directly addresses the therapeutic uncertainty created by a potentially misleading biopsy diagnosis and, we believe, represents the most clinically actionable message of this case. The exposed EFTR approach^12)^ for this case enabled *en bloc* R0 resection despite the lesion’s proximity to the EGJ. A protective mini-laparotomy facilitated secure, tension-free closure of the gastric defect while minimizing postoperative luminal deformity. The final IHC established the diagnosis of GGT, and the local resection by LECS proved both surgically and oncologically sufficient.

Although the great majority of GGTs follow a benign clinical course, malignant variants have been documented.^6,20–22)^ Folpe et al. proposed histological criteria suggestive of malignancy: deep location, tumor size greater than 2 cm, high nuclear grade, atypical mitotic figures, and increased mitotic activity (more than 5 mitoses per 50 HPFs).^21)^ The present tumor demonstrated low mitotic activity (1 per 50 HPFs), a low Ki-67 index (approximately 2%–3%), absence of nuclear atypia, no necrosis, and no vascular invasion, supporting benign biological behavior; despite its size exceeding 2 cm, none of the other malignancy-associated features proposed were present. Nevertheless, long-term surveillance is advisable, because occasional metastatic cases of GGT have been reported.^2,22)^

## CONCLUSIONS

This case provides 4 main educational points. First, NET, GIST, and GGT have fundamentally different therapeutic strategies; therefore, appropriate IHC is essential for accurate differentiation and treatment selection. Surgeons should communicate with pathologists and request an appropriate IHC panel when necessary. Second, when the diagnosis of a gastric SEL remains uncertain, LECS offers an organ-preserving approach by achieving complete local excision while providing an intact specimen for definitive histopathological and IHC evaluation, with additional surgery reserved for cases requiring it based on the final diagnosis. Third, when clinical and pathological findings are discordant, direct communication with the pathologist is essential to resolve diagnostic discrepancies. Fourth, greater awareness of GGT and its distinguishing features among surgeons, endoscopists, and pathologists is needed to reduce diagnostic errors and avoid unnecessary extensive gastric surgery.

## DECLARATIONS

### Availability of data and materials

All data generated or analyzed during this case report are included in this published article.
